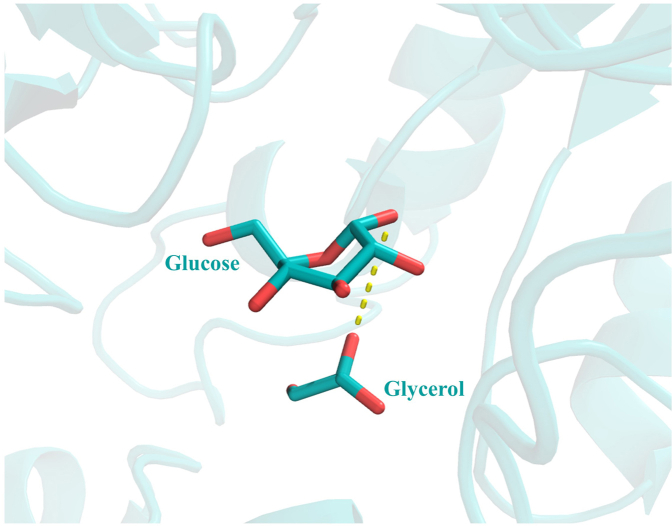# Structural basis of the bifunctionality of *Marinobacter salinexigens* ZYF650^T^ glucosylglycerol phosphorylase in glucosylglycerol catabolism

**DOI:** 10.1016/j.jbc.2024.108127

**Published:** 2024-12-25

**Authors:** Di Lu, Keke Zhang, Chen Cheng, Danni Wu, Lei Yin, Quan Luo, Meiyun Shi, Honglei Ma, Xuefeng Lu

**Affiliations:** 1 School of Chemical Engineering, Marine and Life Sciences, Dalian University of Technology, Panjin, China; 2 Key Laboratory of Biofuels, Qingdao Institute of Bioenergy and Bioprocess Technology, Chinese Academy of Sciences, Qingdao, China; 3 Shandong Energy Institute, Qingdao, China; 4 Qingdao New Energy Shandong Laboratory, Qingdao, China

**Keywords:** glucosylglycerol phosphorylase, GH13_18 family, α-d-glucose-1-phosphate, crystal structure, double displacement mechanism

## Abstract

2-*O*-α-Glucosylglycerol (GG) is a natural heteroside synthesized by many cyanobacteria and a few heterotrophic bacteria under salt stress conditions. Bacteria produce GG in response to stimuli and degrade it once the stimulus diminishes. Heterotrophic bacteria utilize GG phosphorylase (GGP), a member of the GH13_18 family, *via* a two-step process consisting of phosphorolysis and hydrolysis for GG catabolism. However, the precise mechanism by which GGP degrades GG remains elusive. We determined the 3D structure of a recently identified GGP (MsGGP) of the deep-sea bacterium *Marinobacter salinexigens* ZYF650^T^, in complex with glucose and glycerol, α-d-glucose-1-phosphate (αGlc1-P), and orthophosphate (inorganic phosphate) at resolutions of 2.5, 2.7, and 2.7 Å, respectively. Notably, the first αGlc1-P complex structure in the GH13_18 family, the complex of MsGGP and αGlc1-P, validates that GGP catalyzes GG decomposition through consecutive phosphorolysis and hydrolysis. In addition, the structure reveals the mechanism of high stereoselectivity on αGlc1-P. Glu231 and Asp190 were identified as the catalytic residues. Interestingly, these structures closely resemble each other, indicating minimal conformational changes upon binding end-product glucose and glycerol, or the intermediate αGlc1-P. The structures also indicate that the substrates may follow a specific trajectory and a precise order toward the active center in close proximity and in a geometrically favorable orientation for catalysis in a double displacement mechanism.

2-*O*-α-Glucosyglycerol (GG) is a natural glycoside biosynthesized by numerous cyanobacteria and a limited number of heterotrophic bacteria in environments characterized by elevated salinity levels (1, 2). It is also found in some traditional Japanese foods, such as sake, miso, and mirin, as a fermentation product giving a good body taste. GG showcases a myriad of fascinating physicochemical properties and biological functions, encompassing osmoprotection, clear-cut sweetness (0.55-fold that of sucrose sweetness without any bitterness), noncariogenicity, minimal hygroscopicity, protective function on macromolecules, exceptional water retention capabilities, superb biocompatibility, and promising antitumor effects (2, 3, 4, 5, 6). These unique characteristics render it highly valuable for potential applications in cosmetics, health care, food service, enzyme production, and pharmaceuticals (7, 8, 9). Currently, GG is commercially manufactured *via* at least two distinct routes: enzymatic synthesis and native extraction from cyanobacteria (7). Enzymatic production of GG as well as its isomers has been successfully scaled up for industrial application (10, 11). This was achieved either through the utilization of a side reaction catalyzed by a glycoside hydrolase (GH) family 13 enzyme, specifically sucrose phosphorylase (Enzyme Commission [EC] number: 2.4.1.7) (12, 13, 14), as documented in previous research, or employing a transglycosylation reaction catalyzed by a commercially available β-glucosidase (3). GHs constitute a vast group of enzymes with the capacity to catalyze the hydrolysis of glycosidic bonds within carbohydrates. They are ubiquitously found in nature and play pivotal roles in a wide array of physiological and biochemical processes. Several GHs sourced from bacteria or fungi have the ability to catalyze the synthesis and/or breakdown of GGs in *in vitro* systems (8, 15, 16, 17, 18, 19, 20, 21).

BsGGP (EC number: 2.4.1.332) and MaGGP (EC number: 2.4.1.359) are two novel GH family members identified in *Bacillus selenitireducens* and *Marinobacter adhaerens*, respectively (22, 23, 24). Both enzymes exhibit the capacity to catalyze the reversible phosphorolysis of GG and therefore named as GG phosphorylase (GGP). Utilizing the reverse phosphorolysis reaction, GG can be synthesized from glucose-1-phosphate and glycerol using either BsGGP or MaGGP (22, 23, 24). Despite sharing the common designated name as GGP, these enzymes showcase distinct catalytic characteristics. BsGGP catalyzes the synthesis of GG from β-glucose-1-phosphate and glycerol, whereas MaGGP is responsible for converting α-glucose-1-phosphate (αGlc1-P) and glycerol to GG with the preservation of the anomeric configuration. BsGGP is classified within the GH65 group and is believed to catalyze the anomer-inverted reaction through a single displacement mechanism (25), aided by a general acid residue Glu475 (23). MaGGP belongs to the GH13_18 subfamily with retaining glycoside phosphorylases that act on α-glucosides (24). Structurally, GGP in this family is distinguished by a (β/α)_8_-barrel housing the catalytic domain known as domain A. In addition to the catalytic domain, these enzymes typically feature three other domains (Fig. 1*B*) (26). The proposed reaction mechanism for the GH13_18 family involves a double displacement reaction, forming a covalent enzyme–glucosyl intermediate (Fig. 2). The reaction commences with the simultaneous protonation of the ether linkage oxygen atom by the proton donor (Glu231) and nucleophilic attack by Asp190 on the glucosyl moiety's anomeric carbon. This results in the formation of a covalently linked enzyme–substrate intermediate and the liberation of glycerol. Subsequently, the intermediate can interact with phosphate, leading to the eventual release of glucose-1-phosphate. However, the involvement of residue Asp190 in the nucleophilic attack appears less plausible in the enzyme's structure, and the mechanisms governing substrate binding to the pocket and the stereoselectivity of the catalytic reaction remain enigmatic.

**Figure 1:**
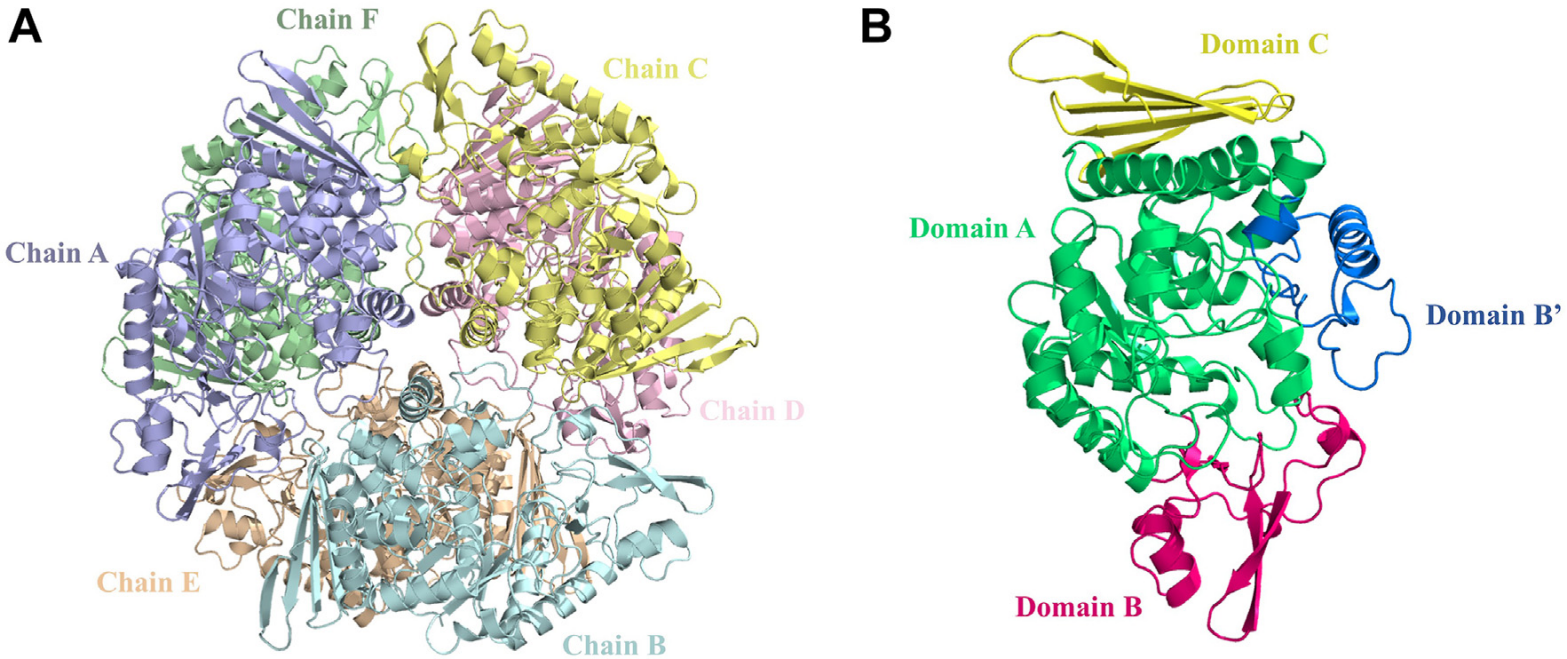
**Schematic representation of the hexameric structure and protomer domain organization of MsGGP.***A*, the hexameric structure complex with glucose and glycerol, chains A, B, C, D, E, and F are colored *light blue, pale cyan, pale yellow, light pink, wheat, pale green*, respectively. *B*, the domain division of MsGGP. Domains A, B, B′, and C are colored *lime green, warm pink, marine*, and tv_*yellow*, respectively. MsGGP, GGP from *Marinobacter salinexigens*.

**Figure 2:**
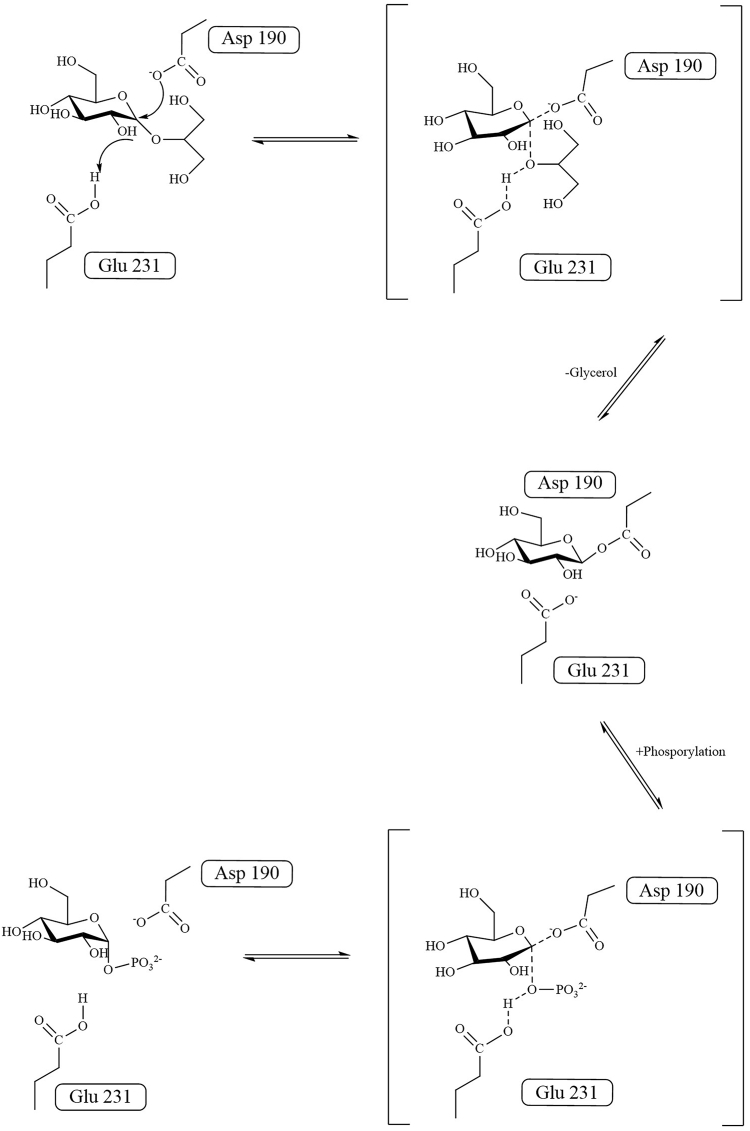
Schematic representation of the degradation mechanism by glucosylglycerol phosphorylase (MsGGP). MsGGP, GGP from *Marinobacter salinexigens*.

Recently, an MaGGP homolog from the deep-sea microorganism *Marinobacter salinexigens* ZYF650^T^, named as MsGGP, was characterized (27). Like MaGGP, MsGGP falls in the GH13_18 subfamily. The purified enzyme was validated to catalyze the breakdown of GG, yielding glycerol and glucose as end products (27). This observation implies that GG hydrolysis occurs in the presence of MsGGP. MsGGP exhibited its peak activity at 45 °C and pH of 6.6, close to the optimal conditions observed for MaGGP (24). *In vitro* analyses demonstrated that MsGGP drives the degradation of GG in a consecutive two-step mechanism involving phosphorolysis and hydrolysis (27). On the other hand, the rational design of MaGGP was undertaken to enhance the enzyme's thermal stability and activity (26). Analysis of the crystal structure and molecular dynamics simulations elucidated the factors contributing to improved thermostability and catalytic efficiency (26). Nevertheless, the catalytic and stereoselective mechanisms of GGP within the GH13_18 family toward GG degradation have not yet been fully elucidated.

In the present study, X-ray crystallography was employed to elucidate the molecular mechanisms underlying GG recognition, phosphorolysis, and subsequent hydrolysis of GGP. Three crystal structures of MsGGP were successfully determined: one in complex with glucose and glycerol (MsGGP–Glc–Gol), one with αGlc1-P (MsGGP–αGlc1-P), and the other one with phosphate (MsGGP–Pi [inorganic phosphate]). These structures, portraying the initial intact complexes of a glucosylglycerol phosphorylase, offer crucial insights into the catalytic mechanism. The findings of this research will further promote biochemical investigations on GGPs to expand our comprehension of the substrate specificity of the GH family enzymes participating in GG degradation and/or biosynthesis.

## Results

### Structure quality of MsGGP–Glc–Gol, MsGGP–**α**Glc1-P, and MsGGP–Pi

This study successfully crystallized the MsGGP protein bound to end products, glucose and glycerol, MsGGP bound to intermediate product, αGlc1-P, and MsGGP bound to Pi (Table 1). We aimed to elucidate the crystal structure of MsGGP–GG; however, our attempts were unsuccessful. MsGGP is identified as a cyanobacteria-distinct pathway for GG degradation in heterotrophic bacteria. It catalyzes GG decomposition through a two-step process involving phosphorolysis and hydrolysis *in vitro* (27). In this context, αGlc1-P is recognized as an intermediate product, whereas glucose and glycerol are considered as the final products. The final models of MsGGP complexed with different ligands exhibit distinct characteristics. The final model of MsGGP–Glc–Gol consists of 2874 amino acid residues distributed among all subunits, along with 29 glycerol molecules and 1205 water molecules, yielding an *R*-factor of 16.71% at a resolution of 2.5 Å. The average thermal factor for the main-chain atoms in this model is 43.43 Å^2^. The final model of MsGGP–αGlc1-P comprises 2874 amino acid residues across all subunits, accompanied by five glycerol molecules and 125 water molecules, resulting in an *R*-factor of 20.23% at a resolution of 2.7 Å. The average thermal factor for the main-chain atoms in this model is 77 Å^2^. Notably, these two models lack the first residue for each subunit. Finally, the final model of MsGGP–Pi comprises 1315 amino acid residues for all subunits, incorporating three phosphate anions, two sulfate anions, and 162 water molecules, leading to an *R*-factor of 16.41% at a resolution of 2.7 Å. However, this model lacks 125 residues, and the average thermal factor for the main-chain atoms remains at 55.51 Å^2^. All models demonstrate high quality, with 99.8% of residues located in the most favorable and additionally allowed regions and only 0.2% in the generously allowed region.

**Table 1:** Data collection and refinement statistics

	MsGGP–Glc–Gol	MsGGP–αGlc1-P	MsGGP–Pi
PDB code	9J24	9J1U	9J25
Resolution range (Å)	48.39–2.5 (2.589–2.5)	21.04–2.72 (2.817–2.72)	49.09–2.744 (2.843–2.744)
Space group	C 1 2 1	P 21 21 21	C 2 2 21
a, b, c (Å)	172.727, 115.065, 182.134	111.83, 178.5, 180.24	107.244, 176.117, 177.369
α, β, γ	90, 100.558, 90	90, 90, 90	90, 90, 90
Unique reflections	120,122 (11,574)	96,529 (9114)	40,064 (2492)
Completeness (%)	99.06 (96.11)	99.00 (94.78)	90.44 (56.93)
Wilson *B*-factor	35.82	65.66	47.17
Reflections used for refinement	120,066 (11,575)	96,434 (9113)	40,013 (2492)
Reflections used for *R*-free	5921 (567)	4609 (420)	1911 (129)
*R*-work	0.1685 (0.2430)	0.2155 (0.3852)	0.2219 (0.2659)
*R*-free	0.2157 (0.3134)	0.2546 (0.4282)	0.2576 (0.3122)
Number of nonhydrogen atoms	24,582	23,371	10,814
Macromolecules	23,124	23,130	10,612
Protein residues	2874	2874	1317
RMS (bonds)	0.012	0.007	0.007
RMS (angles)	1.43	1.03	1.04
Ramachandran favored (%)	97.80	97.13	97.45
Ramachandran allowed (%)	2.20	2.87	2.55
Ramachandran outliers (%)	0.00	0.00	0.00
Rotamer outliers (%)	2.26	0.79	1.55
Clashscore	7.86	9.15	9.59
Average *B*-factor	43.43	77.44	55.36
Macromolecules	43.52	77.45	55.51
Ligands	58.51	102.94	90.99
Solvent	38.70	54.70	36.74
Number of TLS (translation–libration–screw) groups	25	30	11

### Structure subunit

**Fig. S1:**
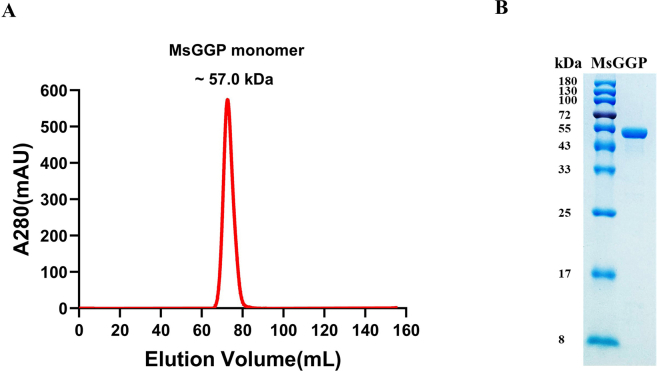


**Fig. S2:**
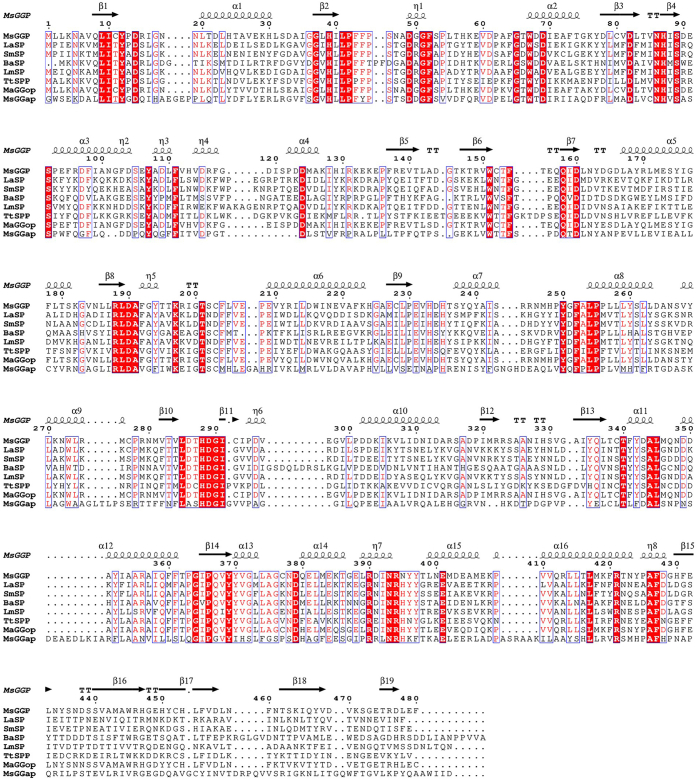


**Fig. S3:**
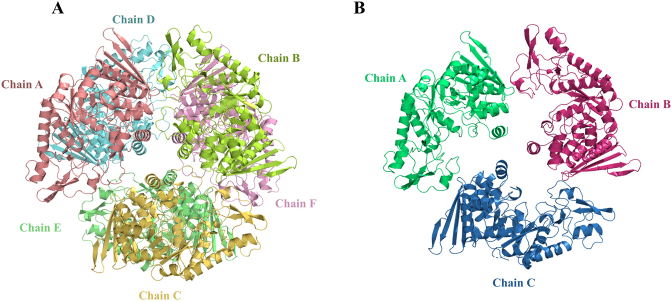


**Fig. S4:**
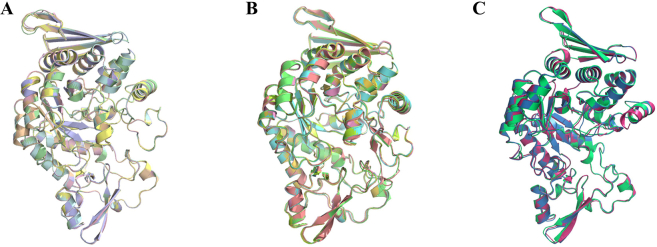


MsGGP, which has been overexpressed in *Escherichia coli* (Fig. S1), has 480 residues per subunit, with a subunit molecular weight of 54,844. It shows high homology to a few characterized members of the GH13_18 subfamily, including MaGGP and sucrose phosphorylases LmSP, LaSP, BaSP, and SmSP (Fig. S2) (8, 24, 28, 29, 30, 31, 32). The overall structure of MsGGP complex is shown in Fig. 1*A* and Fig. S3. The MsGGP–Glc–Gol crystal (*C1 2* 1 space group) contains six protomers (chains A, B, C, D, E, and F) in the asymmetric unit. The hexametric structure of MsGGP–Glc–Gol, illustrated in Fig. 1*A*, was anticipated to exhibit a crystalline packing configuration. The crystal structure of MsGGP, akin to other proteins in the GH13_18 family, consists of four domains: A (residues 1–85, 165–290, and 347–429), B (residues 86–164), B' (residues 291–346), and C (residues 430–480) (Fig. 1*B*). It features a conserved (β/α)_8_-barrel structure typical of GH family 13. The main chain traces of these six protomers exhibit a high degree of similarity, indicated by the RMSD among the Cα atoms in all pairwise comparisons being less than 0.5 Å (Fig. S4*A*).

**Figure 3:**
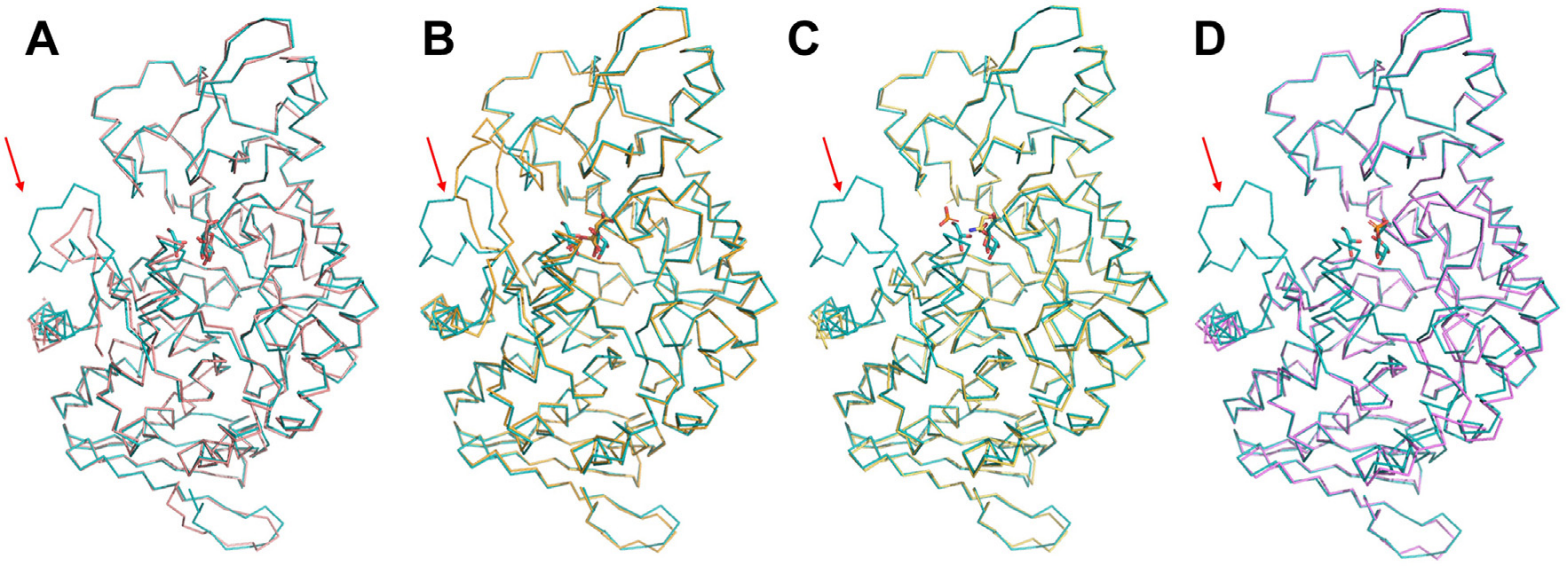
**The superimposition of the subunit of MsGGP–Glc–Gol onto that of MaGGP, MsGGP–αGlc1-P, and MsGGP–Pi.***A*, the superimposition of the subunit of MsGGP–Glc–GoL with MaGGP (Protein Data Bank ID: 7XDQ). *B*, the superimposition of the subunit of MsGGP–Glc–GoL with the subunit bound to αGlc1-P. *C*, the superimposition of the subunit of MsGGP–Glc–GoL with the subunit bound to Tris and Pi. *D*, the superimposition of the subunit of MsGGP–Glc–GoL with the subunit bound to Pi. The subunit of MsGGP–Glc–GoL: *teal*; the subunit of MaGGP: *salmon*; the subunit of MsGGP–αGlc1-P: *bright orange*; the subunit of MsGGP–Tris–Pi: *yellow orange*; and the subunit of MsGGP–Pi: *violet*. The *arrow* shows significantly the conformational changes in these structures. αGlc1-P, α-d-glucose-1-phosphate; MaGGP, GGP from *Marinobacter adhaerens*; MsGGP, GGP from *Marinobacter salinexigens*; Pi, inorganic phosphate.

When the Cα carbon atoms are superimposed between the subunits in MsGGP–Glc–Gol and MaGGP, the average RMSD is 0.47 Å (Fig. 3*A*). The corresponding average RMSD values for the comparisons of MsGGP–Glc–Gol with MsGGP–αGlc1-P, MsGGP–Tris–Pi, and MsGGP–Pi are 0.374 Å, 0.479 Å, and 0.472 Å, respectively (Fig. 3, *B–D*), despite the presence of missing residues in the structure of MsGGP–Pi. These complexes exhibit a high degree of structural similarity among their subunits. By aligning the Cα carbon atoms of the subunits in these three complexes, an average RMSD of less than 0.5 Å is achieved, indicating a fundamental similarity in the subunit structures across these complexes. The high level of structural similarity between the different catalysis states implies minor conformational changes occurring during the catalytic process.

### Active site of MsGGP–Glc–Gol

**Figure 4:**
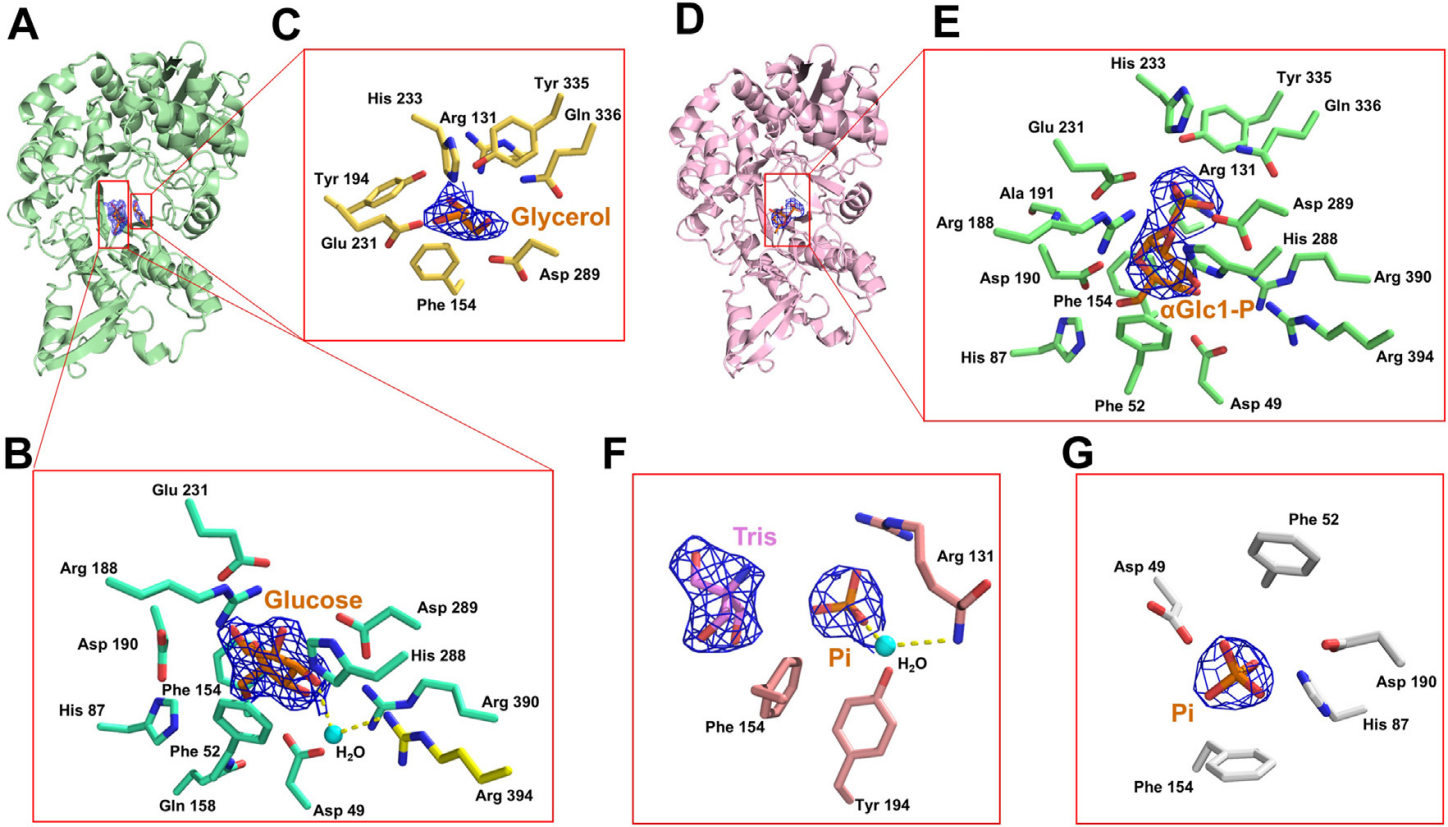
**Close-up view of the MsGGP substrate–product-binding site (*in stereo*).***A*, chain A of MsGGP–Glc–GoL complex. *B*, the key residues within a 4 Å radius of glucose (Glc). *C*, the key residues within a 4 Å radius of glycerol (Gol). *D*, chain E of MsGGP–αGlc1-P complex. *E*, the key residues within a 4 Å radius of αGlc1-P. *F*, the key residues within a 4 Å radius of Pi (chain A, MsGGP–Tris–Pi complex). *G*, the key residues within a 4 Å radius of Pi (chain B, MsGGP–Pi complex). The omit electron density maps of substrates–products are contoured at 3σ. αGlc1-P, α-d-glucose-1-phosphate; MsGGP, GGP from *Marinobacter salinexigens*; Pi, inorganic phosphate.

**Figure 5:**
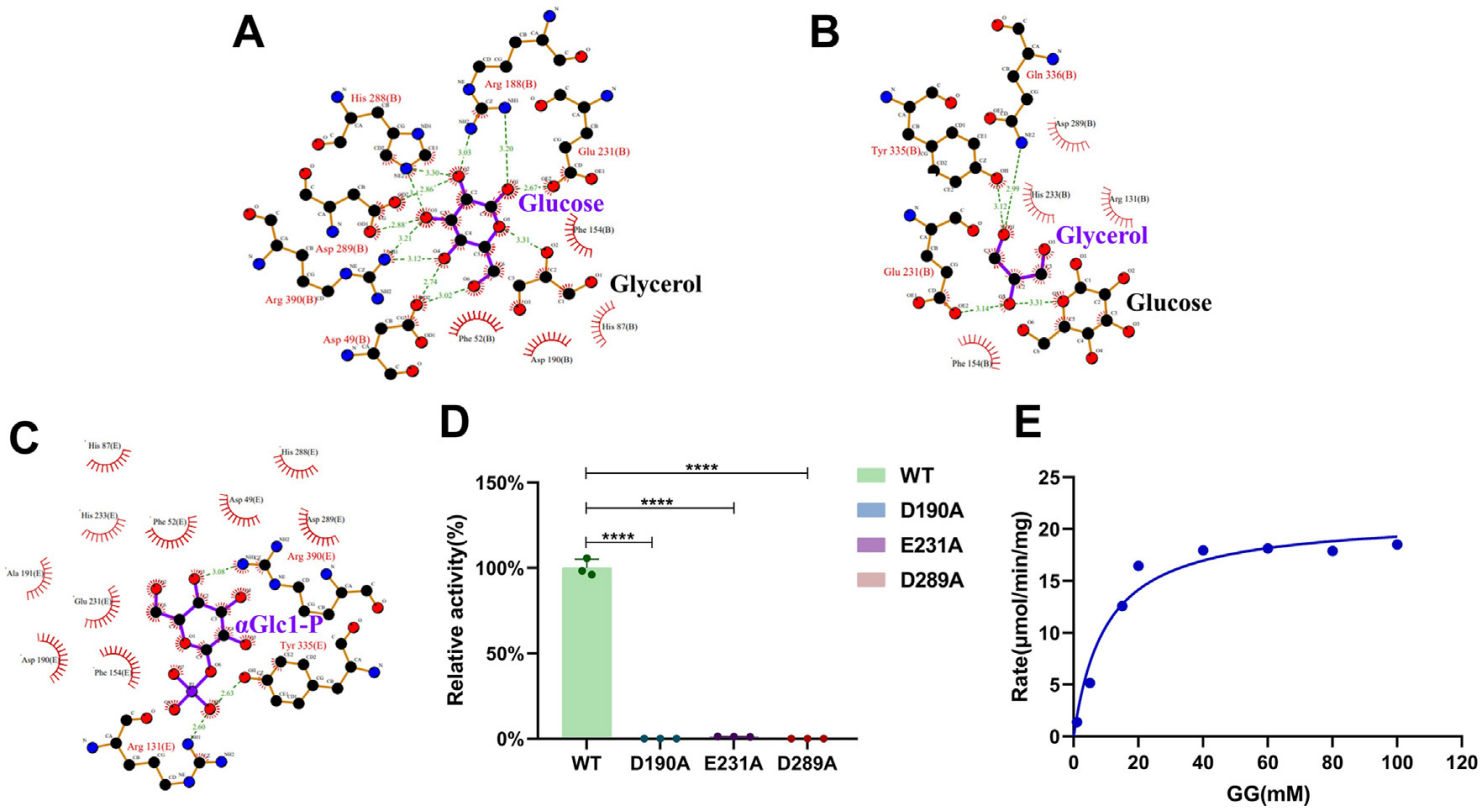
**PDBsum analysis of the residues of active site, mutation analysis, and kinetic detection of MsGGP.***A*, the key residues within a 4 Å radius of Glc. *B*, the key residues within a 4 Å radius of Gol. *C*, the key residues within a 4 Å radius of αGlc1-P. *D*, the enzyme activities of three key residue mutations relative to wildtype one. Each data point represents one experiment shown as a percentage of the WT and plotted as mean ± SD (*bar charts*). Statistical analysis was performed on the non-normalized data using ordinary one-way ANOVA. (∗∗∗∗*p* < 0.0001). *E*, the Michaelis–Menten saturation curve of GG degradation by MsGGP. αGlc1-P, α-d-glucose-1-phosphate; MsGGP, GGP from *Marinobacter salinexigens.*

**Fig. S5:**
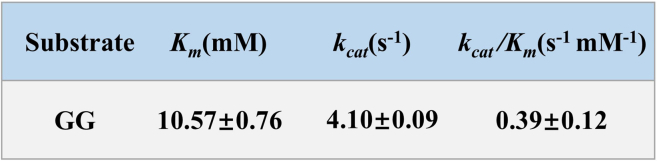


The MsGGP–Glc–Gol crystal (*C1 2 1* space group) contains six protomers (chains A, B, C, D, E, and F) in the asymmetric unit (Fig. 1*A*, Table 1). One subunit in the hexamer interacts with other subunits. Two potential interaction interfaces were identified, with interface areas measuring 1238.6 Å^2^ and 635.2 Å^2^, respectively. However, all the subunit interfaces are distant from the active site and not essential for the catalytic action. Within the asymmetric unit of the MsGGP–Glc–Gol structure, each protomer features an α-glucose and a glycerol molecule occupying the active site, displaying well-defined electron density (Fig. 4, *B* and *C*). Specifically, the glucose molecule is securely situated within the active-site pocket (Fig. 4*A*), closely resembling its positioning in the previously resolved MaGGP structure (26). The interactions of MsGGP and glucose are shown in Fig. 4*B*, Fig. 5*A*, and Table 2. The hydroxyl groups of the glucose have conserved hydrogen-bonding partners. Glucose forms direct hydrogen bonds with the residues Asp49, Arg188, Asp190, Glu231, His288, Asp289, and Arg390 (Table 2). These seven residues stabilize all five hydroxyl groups of glucose and are conserved across all known bacterial GH13_18 family enzymes (Fig. S2). Specifically, Asp49 establishes hydrogen bonds with the hydroxyl groups on C4 and C5 atoms of glucose on the β-face, Asp190 interacts with the hydroxyl groups on C1 atoms on the β-face, Arg390 interacts with the hydroxyl groups on C4 atom on the β-face, His288 interacts with the hydroxyl groups on C3 and C2 atoms on the β-face, Arg188 interacts with the hydroxyl groups on C2 and C1 atoms on the β-face, and Asp289 interacts with the hydroxyl groups on C3 atom on the α-face. Glu231 interacts with the hydroxyl groups on C1 atoms on the α-face. Furthermore, glucose has one indirect hydrogen bond with Arg394 through a water (Fig. 4*B*). Glu231, Asp190, and Asp289 were assumed to be a catalysis residue (26, 33). This was further substantiated by the nearly complete loss of enzyme activity following the mutation of these residues to alanine (Fig. 5*D*). To analyze the properties of MsGGP, the kinetic parameters of the wildtype enzyme were determined for the phosphorylation reaction, with the *K*_*m*_ values and *k*_cat_ assessed at optimal pH and temperature (Fig. 5*E*, Fig. S5). The enzyme exhibited Michaelis–Menten kinetics at the tested substrate concentrations. The *K*_*m*_ value for GG is comparable to that of related glycoside phosphorylases for their natural substrates, which further confirmed the novelty of the specificity of MsGGP. In addition, attempts to analyze the mutant variants of MsGGP revealed that these mutants exhibited minimal activity even when a substantial enzyme mass was used (data not shown).

**Table 2:** The interacting residues and potential hydrogen bonding distances of MsGGP ligands in the protein–ligand complex

	Ligand	Interatomic distance
		Wildtype GGP
Glucose		(Å)
O-1		
	Arg^188^ N-η1	2.8
	Glu^231^ O-ε2	2.6
O-2		
	Arg^188^ N-η2	2.8
	Asp^289^ O-δ2	2.8
	His^288^ N-ε2	3.1
O-3		
	His^288^ N-ε2	3.1
	Asp^289^ O-δ1	2.8
	Arg^390^ N-η1	3.2
O-4		
	Arg^390^ N-η1	3.1
	Asp^49^ O-δ1	3.1
	Asp^49^ O-δ2	2.8
O-5		
	Gol O-2	3.3
O-6		
	Asp^49^ O-δ2	3.0
Glycerol		
O-1		
	Asp^289^ O-δ2	3.4
	Gln^336^ N-ε2	3.4
O-2		
	Glu^231^ O-ε2	3.1
	Glc O-5	3.3
O-3		
	Arg^131^ N-η1	2.8
	Tyr^194^ OH	3.4
	Tyr^335^ OH	3.5
αGlc1-P		
O-1		
	Asp^190^ O-δ2	3.3
O-2		
	His^87^ N-ε2	3.3
	Asp^190^ O-δ2	2.4
O-3		
	Asp^49^ O-δ2	2.4
	Arg^390^ N-η1	3.1
	Arg^390^ N-η2	3.5
O-4		
	Asp^289^ O-δ1	3.3
	Arg^390^ N-η1	3.4
O-5		
	Asp^289^ O-δ2	2.4
O-7		
	Arg^131^ N-η2	3.1
	Asp^289^ O-δ2	3.3
	Gln^336^ N-ε2	3.5
O-8		
	Glu^231^ O-ε2	2.5
	His^233^ N-ε2	3.5
	Asp^289^ O-δ2	3.1
O-9		
	Arg^131^ N-η1	2.6
	Tyr^335^ OH	2.6
Pi (Chain A)		
O-1		
	Tyr^194^ OH	2.6
O-2		
	Tyr^194^ OH	3.2
O-3		
	Arg^131^ N-η1	3.0
	Arg^131^ N-η2	2.8
Pi (Chain B)		
O-1		
	Asp49 O-δ2	3.2
O-2		
	Asp190 O-δ1	3.4
O-3		
	Asp49 O-δ2	3.3
O-4		
	Asp49 O-δ2	3.1

**Figure 6:**
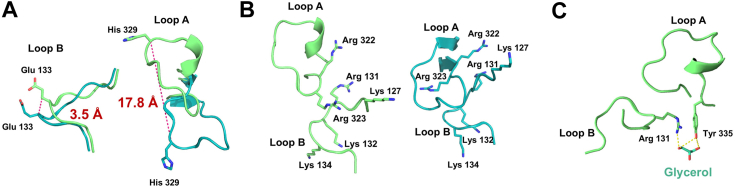
**Structural changes occurring during the enzyme reaction.***A*, close-up view of loops A and B of the end-product glucose and glycerol bound (chain A, *lime*) superimposed on the intermediate αGlc1-P bound (chain E, *teal*), *left*: loop B; *right*: loop A. *B*, *Left*: substrate–product channel formed by loop A and loop B within the structure of MsGGP–Glc–GoL (chain A, *lime*); *Right,* substrate–product channel formed by loop A and loop B within the structure of MsGGP–αGlc1-P (chain E, *teal*). The flexible residues were shown. *C*, the interaction between glycerol and the amino acids Arg131 and Tyr335 involves hydrogen bonding. αGlc1-P, α-d-glucose-1-phosphate; MsGGP, GGP from *Marinobacter salinexigens.*

The interactions of MsGGP and glycerol are shown in Figs. 4*C* and 5*B* and Table 2. The glycerol molecule adopts an extended conformation, and three hydroxyls of the glycerol form hydrogen bonds with the glucose, Arg131, Tyr194, Asp289, Tyr335, and Gln336 (Fig. 4*C* and Table 2). This finding aligns with previous data indicating that performing site-directed mutagenesis on residues Tyr194 and Gln336 in MaGGP leads to a reduction in enzyme activity (24). In the structure of MsGGP, the Tyr335 residue situated within a loop (loop A, see later, Fig. 6) is demonstrated to engage in hydrogen bonding interactions with the O1-hydroxyl group of glycerol, thereby contributing to the stabilization of the glycerol moiety. The O2-hydroxyl of glycerol is located at a distance of 3.0 Å from the O1 atom of glucose. This is a reasonably close distance to represent the acceptor site of glycerol to produce GG with αGlc1-P *via* the retaining phosphorolysis reaction (34). The O2-hydroxyl group of glycerol and the C1 hydroxyl group in glucose are oriented toward residue Glu231 in a coherent manner, establishing a hydrogen bond interaction with Glu231. This orientation is posited to be linked to the retaining phosphorolysis reaction.

**Fig. S6:**
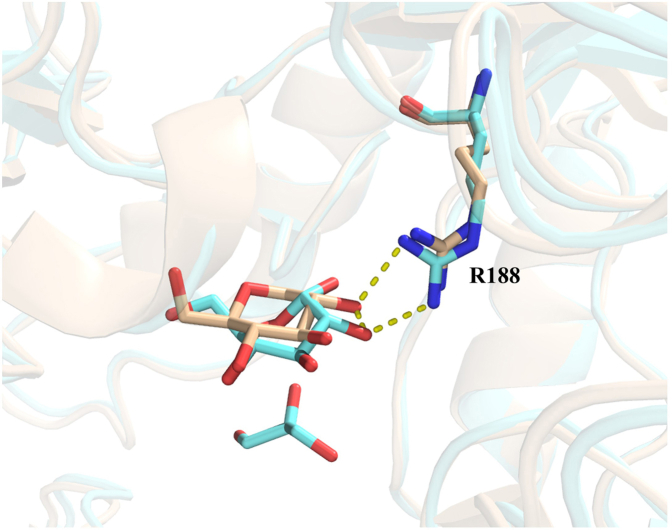


Previously, two structures of GGPs (*i.e.*, BsGGP and MaGGP) have been elucidated, with the former falling under the GH65 family (23) and the latter under the GH family 13 (26). The structure most pertinent for comparison with MsGGP–Glc–Gol is MaGGP. The MaGGP structure (Protein Data Bank [PDB] ID: 7XDQ) features a glucose molecule at the active site that closely overlaps with the one in our structure. The glucosyl moiety in MsGGP displays a slightly twisted α conformation in contrast to that observed in MaGGP. Notably, O4 and O6 are located nearly identically in both structures, whereas O_2_ undergoes a shift of approximately 1.5 Å. Despite this shift, it forms a hydrogen bond with a conserved arginine (Arg188) residue in both structures (Fig. S6).

### Active site of MsGGP–**α**Glc1-P

**Fig. S7:**
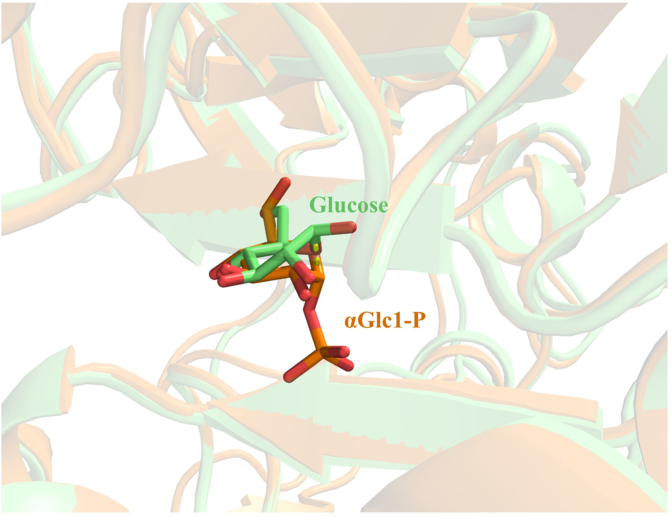


Upon entry into MsGGP, GG undergoes a reaction that poses challenges in obtaining the crystal structure of MsGGP in complex with GG or αGlc1-P. Following extensive experiments and crystal optimization, the structure of MsGGP in complex with αGlc1-P was successfully determined. To the best of our knowledge, this represents the inaugural structure of a phosphorylase complex containing αGlc1-P within the GH13_18 subfamily. The complex structure with αGlc1-P provides validation that GGP facilitates GG decomposition through a two-step process involving phosphorolysis and hydrolysis, as previously reported (27). The MsGGP–αGlc1-P crystal (*P212121* space group) contains six protomers (chains A, B, C, D, E, and F) in the asymmetric unit (Fig. S3*A*, Table 1). In the asymmetric unit of the MsGGP–αGlc1-P structure, two protomers are characterized by the presence of a glucose and a glycerol molecule occupying the active site (subunits B and F), whereas two other protomers showcase an αGlc1-P molecule in the active site (subunits A and E) with clearly defined electron density (Fig. 4, *D* and *E*). Notably, two additional protomers in the unit (subunits C and D) do not exhibit any ligand binding in the active region. The αGlc1-P could be modeled in density but appears slightly crowded because of the narrow binding pocket of MsGGP, suggesting a high degree of substrate selectivity. The overall structure of MsGGP–αGlc1-P closely resembles that of MsGGP–Glc–Gol, as evidenced by an average RMSD of 0.374 (Fig. 3*B*). Within MsGGP–αGlc1-P, the subunit bound to αGlc1-P exhibits a high degree of similarity to the subunits bound to glucose and glycerol, with an average RMSD of 0.369 (Fig. S4*B*). The orientation of the glucosyl moiety in αGlc1-P closely mimics that observed in the MsGGP–Glc–Gol structure, where the plane defined by the glucosyl moiety almost aligns with the corresponding plane in the MsGGP–Glc–Gol structure (Fig. S7). Because of steric hindrance, the phosphate group is oriented downward, resulting in a displacement of approximately 1.6 Å from the anomeric carbon compared with its position in the MsGGP–Glc–Gol structure (Fig. S7).

The glucosyl moiety of αGlc1-P is stabilized by hydrogen bonds through direct interactions with residues Asp49, His87, Arg188, Asp190, Glu231, His288, Asp289, and Arg390 (Figs. 4*E* and 5*C* and Table 2), which align with the glucose in the MsGGP–Glc–Gol structure. In addition, the carbon skeleton of the glucosyl moiety of αGlc1-P is stabilized by hydrophobic interactions with residues Phe52 and Phe154 (Fig. 4*E*). Residues Glu231 and Asp289 also exhibit strong interactions with the glucosyl moiety of αGlc1-P, suggesting their potential roles as catalytic active-site residues based on the spatial arrangement of these residues (Fig. 4*E* and Table 2). However, the crystal structures remained a puzzle at this point. In Fig. 2, Asp190 serves as a general acid by protonating a glycosidic oxygen atom. The distance between glycosidic Glc-O6 and Asp190 O^δ2^ atoms was 5.1 Å in the MsGGP–αGlc1-P complex structure. Because the distance appeared to be too large for a direct protonation, Asp190 would not readily serve as an acid–base catalyst. It is supposed that residues Glu231 and Asp289 may serve as active-site residues catalyzing the reaction, or either Asp190 or the substrate may undergo subtle conformational changes to achieve an appropriate position for catalysis.

The interactions between MsGGP and the phosphate moiety in MsGGP–αGlc1-P are illustrated in Figs. 4*E* and 5*C*, and Table 2. The phosphate group of αGlc1-P is effectively stabilized, oriented toward the exit channel, forming two hydrogen bonds and three salt bridges with Tyr335, Gln336, Arg131, His233, and Arg390. The αGlc1-P molecule within the complex exhibits a twisted conformation, fitting snugly into the narrow pocket of the active site. This orientation of αGlc1-P in MsGGP contributes to its high substrate selectivity and catalytic efficiency. It is significant that Tyr335 and Gln336 directly interact with the phosphate group (Fig. 4*E*), which also interacts with glycerol in the product-bound form of the MsGGP–Glc–Gol structure (Fig. 4*C*).

### Active site of MsGGP–Pi

The crystallographic analysis of wildtype MsGGP complexed with Pi revealed a resolved structure at a high resolution of 2.7 Å (Table 1). The MsGGP–Pi crystal (*C2 2 21* space group) contains three protomers (chains A, B, and C) in the asymmetric unit (Fig. S3*B*). High-quality density maps enable the precise modeling of MsGGP spanning residues 1 to 480, except for regions 311 to 338 and 378 to 391 in chain A, 315 to 336 and 378 to 394 in chain B, and 313 to 338 and 377 to 394 in chain C. All the three protomers in the asymmetric unit of MsGGP–Pi structure contain a phosphate molecule in the active site with a clear electron density (Fig. 4, *F* and *G*). In chain A of the MsGGP–Pi structure (Fig. 4*F*), a distinct electron density corresponding to Tris is observed in the active site, occupying a position similar to that of the glucose molecule in a prior structure (26). Conversely, no such electron density is detected in the other two chains. Notably, the positions of the Pi ions differ significantly among the three chains. In chains B and C, the Pi ions are situated in close proximity to the glucose molecule within the active site, whereas in chain A, there is a slight deviation from the active center because of the presence of Tris occupying the active site. The interactions of MsGGP and Pi are shown in in Fig. 4, *F* and *G*, and Table 2. In chain A, the Pi forms direct hydrogen bonds with Tyr194, Arg131, and Tris (Fig. 4*F*). Specifically, a water molecular mediates hydrogen bond between Pi and the main chain of Arg131 (Fig. 4*F*). In chain B, Pi is stabilized by residues Asp49, Phe52, Asp190, and Phe154, forming direct hydrogen bonds with Asp49 and Asp190 (Fig. 4*F*). Upon comparing chains A and B, it is evident that the active site has the capacity to accommodate both glucose and Pi, facilitating the catalytic transfer of glucosyl to the acceptor molecule. Our hypothesis regarding GG suggests that MsGGP cleaves GG and transfers the glucosyl moiety to Pi, with Pi positioned at the entrance for this purpose.

### Substrate access channel

A plausible mechanism for substrate entry into the fully closed active site of GGP within the GH65 family was previously proposed (23). A similar mechanism has occurred in MsGGP. A comparison of the structures of MsGGP–Glc–Gol and MsGGP–αGlc1-P reveals significant differences in the conformations of two loops, designated as loop A (residues 320–336) and loop B (residues 127–137), with variations of up to 18 Å and 3.5 Å, respectively (Figs. 3*B* and 6*A*). These structural differences are reminiscent of the conformational changes seen in the BiSP protein, which undergoes distinct structural alterations between the covalently bound intermediate and product-bound forms within this region (35). In the structure of MsGGP–Glc–Gol complex, the product-bound form, a channel exceeding 5 Å is formed by loops A and B, which contain flexible lysine or arginine residues (Fig. 6*B*). Within this channel, Tyr335 in loop A and Arg131 in loop B interact with the product glycerol, positioning themselves to regulate its exit (Fig. 6, *C* and *B*). Conversely, in the MsGGP–αGlc1-P complex structure, loop A undergoes a conformational change, approaching loop B. Despite pushing loop B toward the active center, the channel formed by these two loops measures less than 5 Å (Fig. 6*A*). This configuration is considered an open channel in the context of the MsGGP–Glc–Gol complex, facilitating the exit of the product. In contrast, in the MsGGP–αGlc1-P complex, the structure remains in a closed state to promote αGlc1-P hydrolysis. Analysis of the structures of MsGGP–Glc–Gol and MsGGP–αGlc1-P reveals that both loop A and loop B are situated away from the active site. Notably, Tyr335 in loop A and Arg131 in loop B are crucial for product stabilization. These amino acids likely act as sensors to detect reaction completion and subsequently regulate the opening and closing of channels to facilitate either substrate entry or product release.

**Fig. S8:**
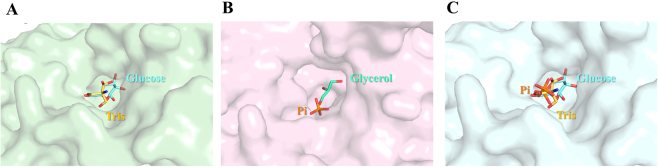


The substrate may follow a specific trajectory toward the active center. In chain A of the crystal structure of MsGGP–Pi, the inactive compound Tris occupies the glucose-binding site within the enzyme's active site (Fig. S8*A*), whereas Pi is positioned at a site corresponding to the glycerol-binding site in the crystal structure of MsGGP–Glc–Gol (Fig. S8*B*). Conversely, in chains B and C of the crystal structure of MsGGP–Pi, Pi occupies the active site of the enzyme (Fig. S8*C*). These observations indicate that Pi likely follows a path leading to the enzyme's active center, serving as a ligand-binding pathway for substrates such as GG or other molecules. Loops A and B are suggested to play a crucial role in regulating the entry and exit of substrates or products. These loops predominantly contain amino acid residues arginine, lysine, glutamic acid, and methionine, known for their highly flexible side chains.

### Docking analysis

The MaGGP crystal structure of the apoenzyme has been previously determined (26), which has high similarity to MsGGP. This study has provided structural information on MsGGP bound to the intermediate product αGlc1-P, MsGGP bound to the final product glucose and glycerol, and the MsGGP bound to Pi.

**Fig. S9:**
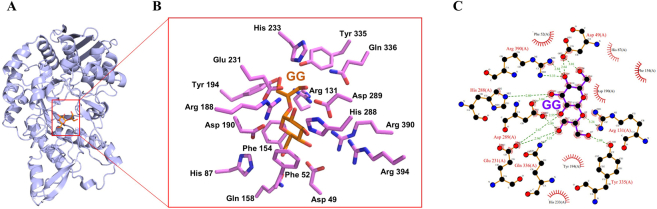


Despite numerous attempts at crystallization, the complex structure of MsGGP bound to GG remains elusive. It is hypothesized that MsGGP either rapidly phosphorylates GG in the presence of phosphate or hydrolyzes GG during crystal growth. We conducted an automated docking analysis to derive plausible binding models of GG. A cluster was identified where the glucosyl moiety of GG was positioned at the same site as glucose, aligning with the structure of MsGGP–αGlc1-P (Fig. S9). The top-ranked cluster exhibited a significantly lower binding energy of −6.4 kcal/mol, surpassing that of other clusters (>6.0 kcal/mol). The glucosyl moiety of GG forms hydrogen bonds with residues Asp49, His87, Arg131, Asp190, Glu231, His288, Asp289, and Arg390 (Fig. S9*B*, Fig. S9*C*), whereas the glycerol moiety engages in hydrogen bonding interactions with the side chains of Arg131, Tyr194, Glu231, Tyr335, and Gln336 (Fig. S9*B*, Fig. S9*C*). Overall, the GG docking complex is stabilized by multiple hydrogen bonds, exhibiting a binding pattern closely resembling that of the MsGGP–αGlc1-P crystal structure.

## Discussion

GG is an important compatible solute identified in many microorganisms and has been commercially produced for diverse applications *via* at least two different biotechnological routes (7, 36, 37). It is primarily biosynthesized by the moderately salt-tolerant cyanobacteria in response to salt stress (7). The processes governing GG anabolism and catabolism have been extensively investigated in cyanobacteria. However, insights into GG metabolism in certain GG-producing heterotrophic bacteria, such as *Marinobacter* and *Pseudomonas*, remain limited. Recently, we have demonstrated the function of *M. salinexigens* GGP (27). It catalyzes GG degradation *via* a two-step process, which is distinct to the known pathway of GG breakdown in cyanobacteria. Our current research has unveiled the mechanism by which GGP breaks down GG. Three crystal structures of the MsGGP complex with glucose and glycerol, MsGGP complex with αGlc1-P, and the MsGGP complex with Pi have been elucidated. Our analysis revealed that glucose, glycerol, and αGlc1-P are situated at the active site of the enzyme, whereas Pi could be found at either the glucose- or the glycerol-binding site. In addition, we have identified the crucial residues within MsGGP—namely Asp49, His87, Asp190, His288, Asp289, and Arg390 (Fig. 4*B*, Table 2)—which stabilize glucose through hydrogen bonding. In contrast, glycerol is stabilized by hydrogen bonds *via* interactions with Arg131, Tyr194, Tyr335, and Gln336 (Fig. 4*C*, Table 2). Pi is a highly dynamic compound that exhibits the capability to bind at sites associated with glucose or glycerol. The stabilization of Pi in these locations is facilitated through robust hydrogen bonding and salt bridge interactions.

Phosphorolysis plays a critical role in the breakdown of GG, with the presence of Pi being essential for efficient degradation (27). MsGGP possesses the ability to utilize either a water molecule or Pi as an acceptor for glycosyl transfer reactions from GG, as evidenced by a prior study (35) and supported by our crystal structure analysis of MsGGP–Glc–Gol. However, the degradation of GG occurs slowly in the absence of Pi (27). Thus, the precise positioning of Pi for phosphorolysis is deemed crucial to initiate the reaction.

**Figure 7:**
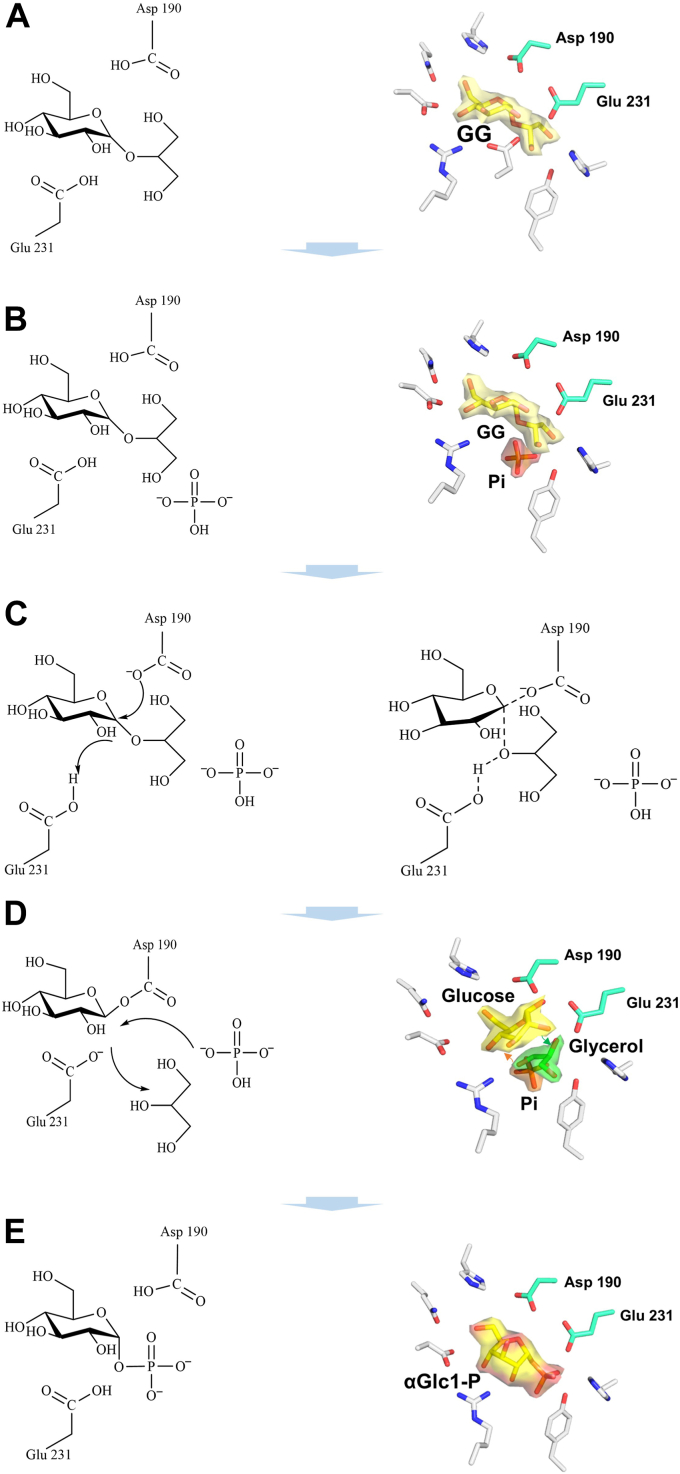
**MsGGP reaction structures.** Putative reaction steps *A*–*E* of MsGGP are shown with corresponding crystal structures at the catalytic center with the formula. The complex structures with GG, GG + Pi, Glc + Gol, and αGlc1-P correspond to schemes A, B, D, and E, respectively. The crystal structures for scheme A and B are mimetic structures. αGlc1-P, α-d-glucose-1-phosphate; MsGGP, GGP from *Marinobacter salinexigens*; Pi, inorganic phosphate.

The initial mechanism involves the transport of GG to the active center (Fig. 7*A*), followed by Pi binding (Fig. 7*B*). This sequential process is corroborated by the structure of the MsGGP–Pi complex, where three subunits were discerned: two subunits exclusively binding to Pi, whereas one subunit binds to Pi alongside Tris in the active site. The presence of Pi in the active site could hinder Tris entry. Upon ingress into the enzyme, GG is stabilized by hydrogen bonds, as demonstrated in the docking model of the GG-bound form. The reaction initiates with the simultaneous protonation of the ether linkage oxygen atom by Glu231 and the nucleophilic attack on the anomeric carbon of the glucosyl moiety by Asp190 (Fig. 7*C*). This leads to the formation of a covalently linked enzyme–substrate intermediate and the release of glycerol, consistent with findings in related enzyme structures (35, 38, 39). Postformation of the covalent intermediate, the glycerol group is liberated and necessitated to move 4 Å away from the C-1 atom of the intermediate (Fig. S10). During this process, glycerol cannot exit the protein because of no conformational changes except for the residues surrounding the active center and the closed channel. Subsequently, the enzyme–substrate intermediate can interact with Pi (Fig. 7*D*), resulting in the liberation of αGlc1-P or further hydrolysis (Fig. 7*E*). In this interaction, glycerol is effectively supported by residues Arg131, Tyr194, and Tyr335, subsequently positioned at a site corresponding to its binding site in the structure of MsGGP–Glc–Gol (Fig. 4*C*). Significant conformational changes occur in loops A and B upon the formation of new glucosidic bond for the product release. The reaction is reversible based on the altered conditions; however, it tends toward degradation reactions (27). This proposed mechanism aligns with the double displacement reaction characteristic of the GH13 enzyme family (40). The presence and precise positioning of Pi may account for the effective breakdown of GG (Fig. 4*F*).

Following phosphorolysis, αGlc1-P undergoes hydrolysis or release from the protein. It is hypothesized that αGlc1-P utilizes a similar mechanism for hydrolysis. Furthermore, given the significance of αGlc1-P as a substrate in cellular processes, phosphorolysis is selected for carbon recycling purposes.

In this study, the precise spatial orientation of amino acids Asp190 and Glu231 is critical for the enzymatic stereoselectivity and retention properties of MsGGP. A concern arises regarding the positioning of Asp190 for catalysis, as it is distant from the anomeric carbon while Asp289 resides in close proximity. It is hypothesized that Asp289, rather than Asp190, may act as the active-site residue catalyzing the reaction, or that either Asp190 or the substrate could undergo subtle conformational changes to achieve the proper position for catalysis. However, the location of Asp289 on the α-face of the glucosyl moiety poses a discrepancy with the anomeric configuration switch in the double displacement mechanism. Comparison of the structures between MsGGP–Glc–Gol and MsGGP–αGlc1-P reveals a displacement of the glucose molecule, repositioning substrate, or the intermediate for the anomeric carbon interaction with Asp190—a finding consistent with another phosphorylase study (41). During the enzymatic reaction, Asp190 and Asp231 interact to form an intermediate with GG, facilitating the cleavage of the glucosyl bond between glucose and glycerol. Specifically, the glucosyl intermediate adopts an inverted configuration with Asp190. Subsequently, Asp231 aids in the transfer of glycerol to a different position, participating in the formation of an intermediate with Pi. Pi then carries out a nucleophilic attack on the α face of the glucosyl intermediate. Our model proposes that Asp289 strongly interacts with GG or αGlc1-P, emphasizing its role as a “transition state stabilizer” in GHs of family GH13—a unified depiction at the molecular level of its impact on catalysis (33).

An essential characteristic of the MsGGP protein is its small binding pocket. The precise positioning of the αGlc1-P substrate within the reaction center is critical as any misalignment results in significant clashes. This clash occurs because the substrate relies on the stabilization provided by multiple hydrogen bonds and salt bridges. In addition to the small binding pocket, residues Glu231 and Asp190 are responsible for modulating the β and α sides of the glucosyl moiety, thereby maintaining the enzyme's retaining function. The structural analysis also reveals that GGP lacks the ability to accommodate β configuration glucosyl derivatives because of steric hindrance.

GGP possesses high specificity to GG. It can catalyze reversible phosphorolysis of GG under proper conditions. This enzymatic activity holds promising implications in biotechnology by enabling the synthesis of GG through the reverse phosphorolytic reaction. GGP presents several advantages, including perfect control of stereoconfigurations, without using any protecting groups, and moderate reaction conditions. MaGGP was genetically modified in a prior study to enhance both its thermostability and catalytic efficiency for glycosylation of polyols (26). It is recognized that the stability and activity of the enzyme are interconnected, and the typical trade-off between them may be mitigated through strategic engineering. Therefore, a comprehensive understanding of the enzyme's mechanism is essential for the development of MsGGP as a versatile enzymatic tool for glycosylation of polyols.

## Experimental procedures

### Strains, plasmids, and chemicals

The expression vectors for N terminally (His)_6_-tagged wildtype GGP from *M. salinexigens* ZYF650^T^ and its mutants were made in our laboratory. Tsingke Biotechnology conducted the sequencing of the plasmid. Our laboratory provided 2-αGG with a purity exceeding 99%, whereas glycerol was procured from China Pharmaceutical Group Chemical Reagent Co, Ltd. Sigma–Aldrich supplied glucose, and Macklin provided alpha-glucose-1-phosphate and kanamycin sulfate. Solarbio was the source of IPTG. The *E. coli* strains BL21(DE3) and DH5α were acquired from Nanjing Vazyme Biotech Co, Ltd. The nickel (Ni)–NTA affinity chromatography column was sourced from Sangon Biotech Co, Ltd.

### Enzymatic activity and kinetic parameter measurement

The activities of MsGGP and its mutants were determined in PB buffer (20 mM KH_2_PO4–K_2_HPO4, pH 6.6) containing 0.05 mg/ml purified enzyme and 20 mM GG. The reactions were carried out at 45 °C for 10 min and stopped by heating at 95 °C for 10 min. One unit of enzymatic activity was defined as the amount of enzyme that catalyzes the production of 1 μmol of glycerol per minute.

To determine the kinetic data of MsGGP on GG, the reaction medium containing Mes buffer (50 mM, pH 6.6), PB buffer (100 mM, pH 6.6), MsGGP (20 μg/ml), and different GG concentrations (1, 5, 15, 20, 40, 60, 80, and 100 mM) was measured at 45 °C for 10 min. *V*_max_ and *K*_*m*_ were calculated by nonlinear regression fitting (saturation hyperbola model) using GraphPad Prism 8.0 (GraphPad Software, Inc).

All quantitative data were presented as means from three independent replicates. All reactions were analyzed using an ICS5000/5000^+^ ion exchange chromatography system (ThermoFisher) with NaOH as the mobile phase.

### Site-directed mutagenesis

Site-directed mutagenesis was performed using a one-step PCR, with the primers detailed in Table S1. The plasmid pET28b-MsGGP, harboring the MsGGP-WT gene served as the template for PCR amplification. Subsequently, the PCR product underwent digestion with the DpnI enzyme at 37 °C for 2 h and was then introduced into *E. coli* DH5α to generate the desired mutant. Following confirmation through DNA sequencing, the mutants were further transformed into *E. coli* BL21 (DE3) for efficient protein expression.

### Sequence alignment

The protein sequences exhibiting significant homology to MsGGP were retrieved *via* the National Center for Biotechnology Information-Blastp function. Protein sequence alignment was performed using MEGA 7 (42), and the secondary structure of MsGGP was superimposed and rendered on the ESPript server (43). The PDBsum website was utilized to investigate the interaction between MsGGP and substrate, identifying amino acids crucial for substrate binding and characterizing their interaction types. Structural overlay through alignment was performed. PyMOL was employed to create all structural images.

### Protein expression and purification

An overnight culture was inoculated at 1% in LB medium supplemented with 50 μg/ml kanamycin and incubated at 37 °C with continuous shaking at 220 rpm. The culture was cultivated until reaching an absorbance of 0.6 to 0.8 at 600 nm, at which point the expression of MsGGP was induced by adding IPTG to a final concentration of 0.2 mM. The protein expression proceeded for 20 h at 18 °C and 180 rpm. The cell pellet was harvested by centrifugation at 6500 rpm for 20 min at 4 °C, then resuspended in Tris–HCl buffer (20 mM Tris, 200 mM NaCl, pH 7.0), and processed using an ATS AH-1500 special high-pressure homogenizer for cell disruption. The homogenizer operated at 800 bars for two cycles to ensure complete cell disruption. Subsequently, the mixture was centrifuged at 10,000 rpm for 1 h at 4 °C to eliminate cell debris. The resulting supernatant was filtered through a 0.22 μm polyethersulfone membrane and applied to an Ni–NTA affinity column for protein purification. Nonspecific proteins were washed out using a gradient of imidazole concentrations (20–80 mM), whereas the adsorbed recombinant protein was eluted with 200 mM imidazole. The protein absorbance at 280 nm was determined using a Nanodrop ND-1000 (Thermo Scientific), and the protein concentration was quantified with the extinction coefficient calculated by the ProtParam tool from the ExPASy server. Finally, the molecular weight and purity were assessed *via* SDS-PAGE (10% gel).

### Cryst allization

In the crystallization experiments, the MsGGP sample, eluted *via* Ni column affinity chromatography, was concentrated to 1 ml through ultrafiltration. Subsequently, it underwent further purification using a HiLoad 16/60 Superdex 200 column (GE Healthcare) with a buffer solution comprising 20 mM Tris–HCl, 200 mM NaCl, and pH 7.0 for size-exclusion chromatography. The purified protein, concentrated to approximately 5 to 15 mg/ml by measuring absorbance and extinction coefficient at 280 nm, was either directly utilized in crystallography experiments or promptly frozen in liquid nitrogen and stored at −80 °C for future use. Protein crystallization was achieved through sitting drop diffusion at 20 °C, employing a commercial high-throughput screening kit and a nanoliter titration Mosquito robot for the experimental process. Crystals of MsGGP–Glc–Glo, MsGGP–Pi, and MsGGP–αGlc1–P were obtained in solutions containing 1.6 M sodium chloride, 8% w/v polyethylene glycol 6000, 20% v/v glycerol; 1.8 M ammonium sulfate, 0.1 M Bis–Tris (pH 6.5), 2% polyethylene glycol monomethyl ether 550; 0.1 M sodium chloride, 0.1 M Bis–Tris propane (pH 9.0), and 25% w/v polyethylene glycol 1500, respectively.

### Data collection and structure determination

Prior to X-ray data collection, the crystals were ﬂash-cooled in liquid nitrogen using 30% (v/v) glycerol as a cryoprotectant. Data collection took place at sector BL19U1, BL18U1, and BL10U2 beamlines of SSRF, with diffraction data for all complex crystals gathered at slightly varying wavelengths as detailed in Table 1. Processing of all datasets utilized HKL-3000 (44), XDS (45), in conjunction with Scalepack2mtz from the CCP4 suite (46). Molecular replacement to solve the phase problem employed an AlphaFold model (47). Residue-by-residue estimates of uncertainty guided the removal of low-confidence residues. A preliminary model was created through collaborative efforts of Phenix and Coot (48), with successive refinement facilitated by the Phenix refinement program (49) and iterative manual adjustments in Coot to achieve specified *R*-factor and *R*-free values as shown in Table 1. Further, the structures of the MsGGP–αGlc1–P and MsGGP–Pi were deciphered using molecular replacement with the MsGGP–Glc–Gol structure as a template. Refinement of the initial model was carried out using Refmac (50) and Phenix refine programs, with construction of the αGlc1-P or Pi model guided by the difference in the electron density map in Coot. Multiple cycles of manual model building and refinements were conducted to enhance the complex structures to attain satisfactory *R*-factor and *R*-free values.

### Molecular docking

The molecular docking analysis between MsGGP and GG was carried out using AutoDockTool, v1.5.6 software (51). Prior to docking, water molecules in MsGGP–αGlc1-P (PDB ID: 9J1U) were eliminated with PyMOL. The X-ray crystal structure containing the entire αGlc1-P molecule (PDB ID: 9J1U) served as the macromolecule, whereas GG was utilized as the ligand for docking. In the composite model of MsGGP-GG, the center grid box was positioned at coordinates center_x = −20.159, center_y = 9.463, and center_z = −23.610. Subsequently, a docking receptor map file encompassing the active-site pocket space was generated. The experiment was iterated 10 times, resulting in nine molecular conformations. These conformations were compared against the glucose moiety in the experimental MsGGP protein structure, and the model that exhibited the best agreement with the experimental data was chosen for structural interpretation.

## Data availability

The coordinates and structure factors of MsGGP–Glc–Gol, MsGGP–αGlc–1P, and MsGGP–Pi structures have been deposited in PDB with PDB IDs 9J24, 9J1U, and 9J25, respectively. The authors declare that the main data supporting the findings of this study are available within the article and its supporting information.

## Supporting information

This article contains supporting information.

## Conflict of interest

The authors declare that they have no conflicts of interest with the contents of this article.

## Figures and Tables

**Fig. S10:**