# Supplementary material for: Structural basis of the bifunctionality of Marinobacter salinexigens ZYF650T glucosylglycerol phosphorylase in glucosylglycerol catabolism
Source: J Biol Chem. 2024 Dec 25;301(2):108127. doi: 10.1016/j.jbc.2024.108127 (PMC11787520; doi:10.1016/j.jbc.2024.108127)
Supplement: Supporting Information [file mmc1.docx]

**Supporting information**

Fig. S1 Size-exclusion chromatographic profile of MsGGP. A. MsGGP is filtered through size exclusion chromatography (SEC) with Hiload^TM^ 16/600 Superdex 200 pg column (GE Healthcare). B. The protein is verified by SDS-polyacrylamide gel electrophoresis.

Fig.S2 Sequence alignment of MsGGP and other proteins from GH13_18. LaSP: *Lactobacillus acidophilus* Sucrose phosphorylase(Q7WWP8); SmSP: *Streptococcus mutans* Sucrose phosphorylase(P10249); BaSP: *Bifidobacterium adolescentis* Sucrose phosphorylase(A0ZZH6); LmSP: *Leuconostoc mesenteroides* Sucrose phosphorylase(Q59495); TtSPP: *Thermoanaerobacterium thermosaccharolyticum* Sucrose 6(F)-phosphate phosphorylase(D9TT09); MaGGoP: *Marinobacter adhaerens* Glucosylglycerol phosphorylase(E4PMA5); MsGGaP: *Meiothermus silvanus* Glucosylglycerate phosphorylase (D7BAR0). Red background indicates sequence identity; red letters indicate sequence similarity. Protein sequence alignments were performed using MEGA 7.0. Drawing was carried out using ESPript 3.

Fig. S3 Schematic representation of the hexameric structure of MsGGP-αGlc1-P (A) and trimeric structure of MsGGP-Pi (B)

Fig. S4 Comparison of each subunit of MsGGP A. MsGGP-Glc-GoL (6 chains). B. MsGGP-αGlc1-P (6 chains). C. MsGGP-Pi (3 chains).

Fig. S5 The kinetic parameter of MsGGP.

Fig. S6 The comparison between MsGGP and MaGGP in the context of the active site. The C2 of glucose undergoes a shift of approximately 1.5 Å, both of the hydroxyl groups of the C2 form a hydrogen bond with the conserved Arg188 residue. MsGGP: Cyan, MaGGP: wheat.

Fig.S7 The comparison between MsGGP-Glc-Gol and MsGGP-αGlc1-P in the context of the active site. The glucosyl moiety almost overlaps with the phosphate group oriented downward. The anomeric carbon of MsGGP-αGlc1-P a displacement of approximately 1.6 Å from that of MsGGP-Glc-Gol. MsGGP-Glc-Gol: limegreen, MsGGP-αGlc1-P: orange.

Fig.S8 The comparison among MsGGP-Glc-Gol, MsGGP-αGlc1-P and MsGGP-Pi in the context of the active site. A. The spatial positions of Tris and Glc in the structures of (MsGGP-Pi, chain A) and MsGGP- Glc GoL (MsGGP-Glc-Gol, chain A). B. The spatial positions of Pi and Gol in the structures of MsGGP-Pi (chain A) and MsGGP-Glc-GoL (chain A). C. The spatial positions of Tris, Pi and Glc in the structures of MsGGP-Pi (chain A,chain B) and MsGGP-Glc-Gol (chain A).

Fig.S9 Docking analysis of GG interacting with MsGGP. A. A GG docking structure of MsGGP based on the structure of MsGGP-αGlc1-P. B. The key residues within a 4 Å radius of GG in stereo. C. The key residues within a 4 Å radius of GG by PDBsum analysis.

Fig.S10 The spatial distance between the anomeric carbon hydroxyl group of Glc and the C2 hydroxyl group of Gol in the end-product structure of MsGGP-Glc-GoL.

Table.S1 Primers used for site-directed mutagenesis

| Primers | Sequences (5'→3') |
| --- | --- |
| D190A-F | GCCTG**GCG**GCGTTTGGCTATACCACCAAAC |
| D190A-R | ACGC**CGC**CAGGCGCAGCAGATTCACGCC |
| E231A-F | TGCCG**GCG**GTGCATGATCATACCAGCTATC |
| E231A-R | TGCAC**CGC**CGGCAGGCATTCCGCGCCGTG |
| D289A-F | CCAT**GCG**GGCATTTGCATTCCGGATGTG |
| D289A-R | TGCC**CGC**ATGGGTATCCAAAACGGTCAC |
